# Artemisinin-Resistant *Plasmodium falciparum* K13 Mutant Alleles, Thailand–Myanmar Border

**DOI:** 10.3201/eid2208.160004

**Published:** 2016-08

**Authors:** Mikael Boullé, Benoit Witkowski, Valentine Duru, Kanlaya Sriprawat, Shalini K. Nair, Marina McDew-White, Tim J.C. Anderson, Aung Pyae Phyo, Didier Menard, François Nosten

**Affiliations:** Mahidol University Faculty of Tropical Medicine, Mae Sot, Thailand (M. BoulléSriprawat K, A.P. Phyo, F. Nosten);; Institut Pasteur in Cambodia, Phnom Penh, Cambodia (B. Witkowski, V. Duru, D. Menard);; Texas Biomedical Research Institute, San Antonio, Texas, USA (S.K. Nair, M. McDew-White; T.J.C. Anderson; F. Nosten);; University of Oxford Nuffield Department of Medicine, Oxford, UK (F. Nosten)

**Keywords:** Malaria, parasites, *Plasmodium falciparum*, K13 mutant alleles, genotype, artemisinin resistance, parasite clearance half-life, ring-stage survival assay, Thailand, Myanmar, antimicrobial resistance

**To the Editor:** Artemisinin resistance (ART-R) in *Plasmodium falciparum* phenotypes may have evolved independently in various areas of the Greater Mekong Sub-region (*1*,*2*), prompting the World Health Organization to change its regional policy from containment to elimination (*3*). Risks associated with ART-R include compromised use of artemisinin combined therapy, partner drug resistance selection, total ART-R resistance, and geographic extension to other malaria-endemic regions (*2*,*3*). Characterization of ART-R in each setting and rapid update of listed phenotypes classified as in vitro resistant to this antimalarial drug are needed.

Detected in western Cambodia in 2008, ART-R has been observed in neighboring countries, notably at the Thailand–Myanmar border (*4*,*5*). Resistance is partial and manifests by an increased parasite clearance half-life (PCHL) of >5 hours in patients receiving artemisinin monotherapy or artemisinin combined therapy (*6*). In vitro, ART-R phenotype has been characterized by the ring-stage survival assay (RSA^0–3h^, cutoff 1%) (*7*) and mutations in the propeller domain of a *kelch* gene on chromosome 13 (K13) (*8*,*9*). However, tremendous K13 variability in different genetic backgrounds requires confirmation of specific alleles as ART-R markers (*2*,*3*); even statistically significant clinical associations are rarely unequivocal (*5*–*9*).

On the Thailand–Myanmar border where ART-R has been documented (*4*), we investigated K13 mutations in clinical and in vitro phenotypes. Patients with nonsevere *P. falciparum* hyperparasitemia infections treated during 2011–2013 at the Shoklo Malaria Research Unit (Mae Sot, Thailand) were treated with artesunate, then combined artesunate/mefloquine (*5*). We retrospectively selected 33 case-patients on the basis of PCHL outcome to analyze a broad parasite clearance distribution with available cryopreserved isolates. Full written consent from all patients was obtained. PCHL was calculated on the basis of initial and repeated parasitemia measurement every 6 hours until undetectable asexual parasitemia (*6*) was achieved. Venous blood samples were cryopreserved before drug administration (day 0).

Short-term, culture–adapted parasites (3% hematocrit; RPMI-1640 supplemented with 10% human serum, 0.05 mg/mL hypoxanthine, 2 mg/mL sodium bicarbonate, 2 mg/mL glucose, 0.04 mg/mL gentamicin, 0.3 mg/mL L-glutamine in a 37°C candle-jar atmosphere) were split for blinded RSA^0–3h^ and K13 genotyping. We performed RSA^0–3h^ in duplicate by selecting early rings (0–3 h) in a combination of percoll gradient and sorbitol lysis, followed by a 6-h exposure to 700 nmol/L dihydroartemisinin (*7*). RSA survival rate was measured microscopically 66 hours after drug removal and calculated as the quotient of parasitemia upon DHA exposure over control parasitemia with dimethyl sulfoxide. Only 25 isolates that were successfully culture-adapted provided RSA survival rates.

After the phenotypical assays, the genotypes were obtained and K13 regions were amplified by using 3 primer sets: fragment 1 (1725380–1725680 bp, pos 211–302), F-tgaaaatatggtaggtgatt and R-atcgtttcctatgttcttct; fragment 2 (1725980–1726520 bp, pos 419–570), F-atctaggggtattcaaagg, R-ccaaaagatttaagtgaaag; and fragment 3 (1726400–1726940 bp, pos 545–707), F-ctgccattcatttgtatct, R-ggatatgatggctcttcta) before sequencing (*8*). The 33 monoclonal isolates yielded clear K13 gene sequences. All except 4 isolates from patients who had PCHL >5 h had a single K13 mutant allele (19/23), and all isolates except 1 (G538V) from case-patients who had PCHL <5 h carried the K13 3D7 wild-type allele (9/10). PCHL was significantly different between K13 wild-type (n = 13, median 4.3 h) and mutant (n = 20, median = 7.2 h) alleles (p<0.01 by Mann-Whitney U test). Among the 25 isolates successfully tested, RSA survival rates differed significantly between K13 wild-type (n = 10, median 0.5%) and mutant (n = 15, median 3.5%) alleles (p<0.001 by Mann-Whitney U test). When PCHL was present <5 h, RSA survival rates (n = 7, median 0.5%) were significantly lower than when PCHL was >5 hours (n = 18, median 3.1%) (p = 0.001 by Mann-Whitney U test).

In detail (Figure), C580Y and N458Y mutants were consistently associated with PHCL >5h and RSA values >1%. The C580Y allele has been repeatedly confirmed as a molecular marker of ART-R (*5*,*7*–*9*). Previous reports have inconsistently associated the N458Y mutation with ART-R; 7 case-patients with PCHL >5 h were reported by Ashley et al. (*5*), and 1 artemisinin sensitive case was reported at the China–Myanmar border (*10*). Nevertheless, this mutation has not been confirmed in vitro (*3*). We confirmed the mutation in vitro, and in vivo, according to the World Health Organization definition (*3*), this K13 allele as a molecular marker of ART-R.

**Figure:**
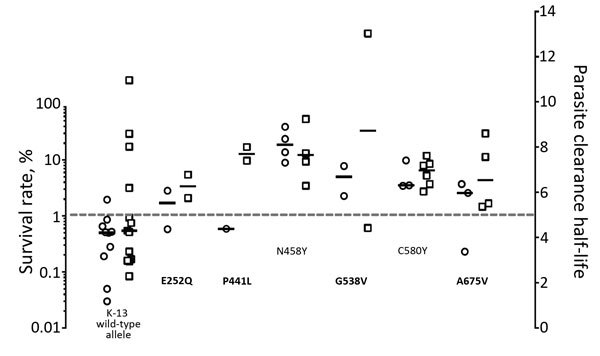
Distribution of parasite clearance half-lives (n = 33, squares) and Ring-stage survival assay survival rates (n = 25, circles) of *Plasmodium falciparum* isolates from patients on the Thailand–Myanmar border, determined on the basis of each K13 genotype. Mean survival rate of duplicate measures are showed for each isolate. Dashed line represents the cutoff value for parasite clearance half-life (artemisinin resistance >5 h) and RSA survival (artemisinin resistance >1%). K13 alleles N458Y and C580Y were consistently associated with parasite clearance half-life and survival rates above threshold. Bold text indicates K13 alleles with variable parasite clearance half-life and RSA associations. Horizontal bars represent median values for each K13 genotype. Survival rate for laboratory reference 3D7 strain was 0.2%.

Conflicting data observed between PCHL and RSA values for 4 mutant alleles (E252Q, P441L, G538V, and A675V) require further targeted approaches to relate them to previous reports. In a study in which only PCHL were reported (*5*), the proportion of slowly clearing infections were 69%, 0%, 30%, and 61% for the P441L, E252Q, G538V, and A675V alleles, respectively. Discrepancies can result from confounding pharmacologic (drug level, partner drug), immunologic, and parasitologic (genetic background, parasitic stage at treatment initiation) factors.

RSA results and K13 genotypes were associated with delayed parasite clearance, emphasizing the pertinence of each method to define ART-R. In this area, N458Y is a marker of ART-R. To solve conflicts about specific mutations, more detailed characterization in vitro and in vivo is needed.
